# Atomically Resolved Defects Modulate Electronic Structure in Plasma-Assisted 2D Janus MoSSe Monolayers

**DOI:** 10.1021/acsnano.5c14446

**Published:** 2025-12-09

**Authors:** Zi-Liang Yang, Yu-Chieh Lin, Mayur Chaudhary, Li-Sheng Lin, Chih-Yang Huang, You-Jie Lin, Jyh-Pin Chou, Li-Chyong Chen, Kuei-Hsien Chen, Yu-Lun Chueh, Ya-Ping Chiu

**Affiliations:** † Graduate School of Advanced Technology, 33561 National Taiwan University, Taipei 10617, Taiwan; ‡ Department of Physics, 33561 National Taiwan University, Taipei 10617, Taiwan; § Center of Atomic Initiative for New Materials, 33561 National Taiwan University, Taipei 10617, Taiwan; ∥ Department of Materials Science and Engineering, National Tsing-Hua University, Hsinchu 30013, Taiwan; ⊥ Center for Condensed Matter Sciences, 33561 National Taiwan University, Taipei 10617, Taiwan; # Institute of Atomic and Molecular Sciences, 38017 Academia Sinica, Taipei 10617, Taiwan; ∇ College of Semiconductor Research, National Tsing-Hua University, Hsinchu 30013, Taiwan; ○ Department of Physics, National Sun Yat-sen University, Kaohsiung 80424, Taiwan; ◆ Department of Materials Science and Engineering, Korea University, Seoul 02841, Republic of Korea

**Keywords:** Janus, 2D materials, scanning tunneling microscopy, STM, defect, electronic structure, electronic device

## Abstract

Janus transition metal dichalcogenides, such as MoSSe, are potential materials for advanced electronics, yet their real-world device performance often fails to meet theoretical expectations. The origin of this discrepancy, rooted in atomic-scale imperfections, has remained critically unexplored. Here, using scanning tunneling microscopy and spectroscopy, this work provides atomic-scale insights into the complex electronic structures of monolayer Janus MoSSe, revealing distinct defect species that govern device performance. The residual sulfur dopants are found to introduce a broad band (≈0.5 eV) of shallow in-gap states near the valence band with spatially inhomogeneous distribution. Moreover, this work unveils two distinct native charge defects with spatially electronic influence extending ≈2.5 nm: conductive charge traps that reduce the local effective bandgap by more than half and insulating scattering centers that impede carrier transport. This microscopic understanding of defect-induced electronic modifications explains how atomic-scale imperfections influence macroscopic device limitations, providing fundamental design criteria for the engineering of Janus devices.

## Graphical abstract

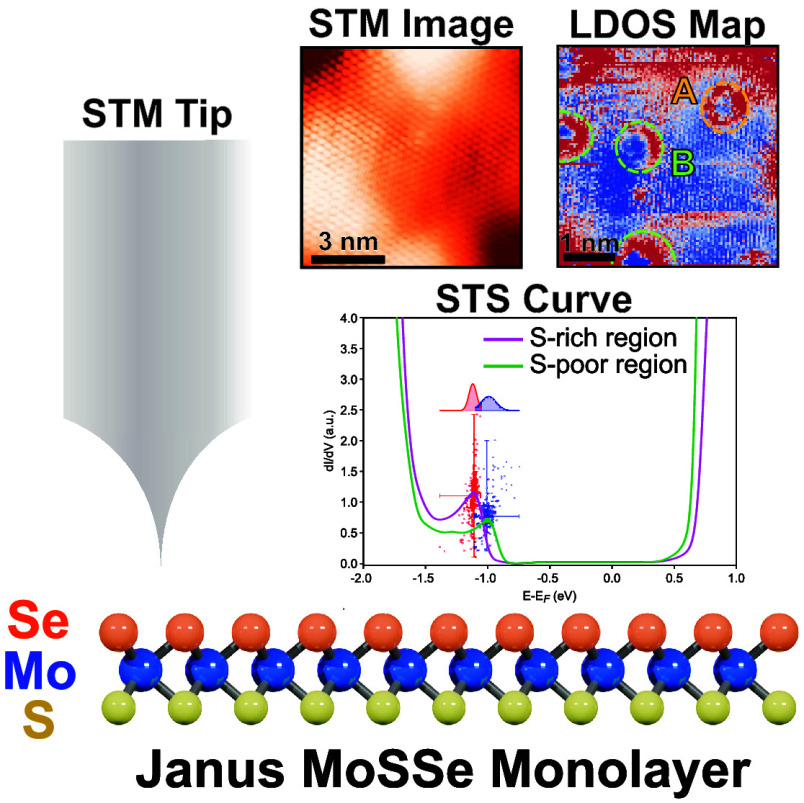

The fundamental scaling limits of silicon-based electronics have fueled an urgent search for alternative materials. Two-dimensional (2D) materials, particularly the semiconducting transition metal dichalcogenides (TMDs), such as MoS_2_ and WSe_2_, have emerged as leading candidates for postsilicon electronics owing to their atomic thickness, inherent band gaps, and exceptional electronic and optoelectronic properties.^1–4^ While promising for ultrathin transistors, photodetectors, and flexible electronics, the performance of devices based on conventional, symmetric TMDs is often constrained by factors such as low mobility,^5^ low operating current, and high contact resistance.^6–8^ This motivates the exploration of new 2D materials with enhanced or distinct functionalities.

A promising strategy to overcome these limitations is to intentionally break the out-of-plane mirror symmetry inherent to conventional TMDs intentionally. This has led to the recent synthesis of “Janus” TMD monolayers, such as MoSSe and WSSe, which feature different chalcogen atoms on opposite faces of the transition metal layer.^9–13^ This structural asymmetry gives rise to a permanent, out-of-plane electric dipole. This intrinsic dipole gives rise to a range of unique properties predicted by theory and increasingly verified by experiments.^14–17^ For instance, Janus TMDs exhibit strong out-of-plane piezoelectricity and a significant Rashba effect,^18–20^ making them highly attractive for nanoelectromechanical systems and spintronics,^21–24^ respectively. Furthermore, the built-in field facilitates ultrafast, directional charge transfer in van der Waals heterostructures,^17,25–27^ leading to photovoltaic devices with power conversion efficiencies that dramatically surpass those based on symmetric TMDs,^28,29^ and also makes Janus materials promising for applications in catalysis,^30,31^ chemical sensing,^32,33^ and neuromorphic computing.^34^ Recently, the wafer-scale synthesis of monolayer Janus MoSSe and WSSe has been demonstrated, paving the way for industry applications.^9^ These attributes position Janus MoSSe as a potentially promising material platform for future electronic and optoelectronic applications compared to conventional MoS_2_.^15,35,36^

Despite this immense promise, a critical aspect remains largely unexplored: the local electronic structure of realistic, synthesized Janus MoSSe at the atomic scale.^37^ The performance of 2D materials is dictated not by the properties of a perfect crystal but by the complex landscape of inevitable imperfections.^38–40^ Atomic-scale defects, such as chalcogen vacancies, are known to introduce localized in-gap electronic states that can act as charge traps, scattering centers, or nonradiative recombination sites, profoundly impacting device mobility, efficiency, and reliability.^39,41^ Moreover, a microscopic understanding of these imperfections is equally critical for emerging applications, as defect sites can be engineered to serve as active catalytic centers,^42–44^ sensitive adsorption sites for chemical sensors,^45,46^ or functional charge-trapping states for neuromorphic devices.^47–50^ Consequently, a fundamental understanding of the electronic structure of Janus MoSSe must include a rigorous investigation of its defect physics. To date, while macroscopic probes such as angle-resolved photoemission spectroscopy (ARPES) and photoluminescence (PL) have characterized the average band structure of Janus MoSSe,^37,51^ a direct, atomic-scale experimental study is absent from the literature, leaving a critical gap between the predicted potential of ideal MoSSe and the reality of its application.

In this study, we use scanning tunneling microscopy and spectroscopy (STM/S) to conduct a comprehensive, atomic-scale investigation of the electronic structure of the synthesized monolayer Janus MoSSe. Our experiments uncover a complex defect landscape dominated by distinct types of native imperfections. First, we observe that residual sulfur dopants induce a broad band of in-gap states. The inhomogeneous distribution of these dopants further creates a complex electronic background, where the in-gap states are demonstrably shallower and more intense in sulfur-rich regions. Furthermore, we discover and characterize two functionally distinct charge defects with spatially electronic influence extending ≈2.5 nm: a Type A defect that acts as a conductive charge trap by locally reducing the bandgap by more than half, and a Type B defect that has a lower local density of states (LDOS) compared to the surrounding material, serving as an insulating scattering center. The study provides the experimental benchmark for the nanoscale electronic properties of Janus MoSSe, offering a direct explanation for its performance limitations and a guide for future materials engineering.

## Results and Discussion

In this work, STM is used to unveil the electronic structure of monolayer Janus MoSSe on an atomic scale. Monolayer Janus MoSSe was synthesized from CVD-grown MoS_2_ via a plasma-assisted selenization process,^9^ as shown in Figure 1a. This process utilizes a low-pressure H_2_ plasma environment at an elevated temperature of 200 °C to selectively desorb sulfur atoms from the top layer of MoS_2_, which are subsequently replaced by selenium atoms introduced upstream. A suite of characterization techniques was then employed to confirm the successful synthesis. The details of the Janus conversion process can be found in the Experimental Section, our previous work,^9^ and Supplementary Note 1.

**Figure 1:**
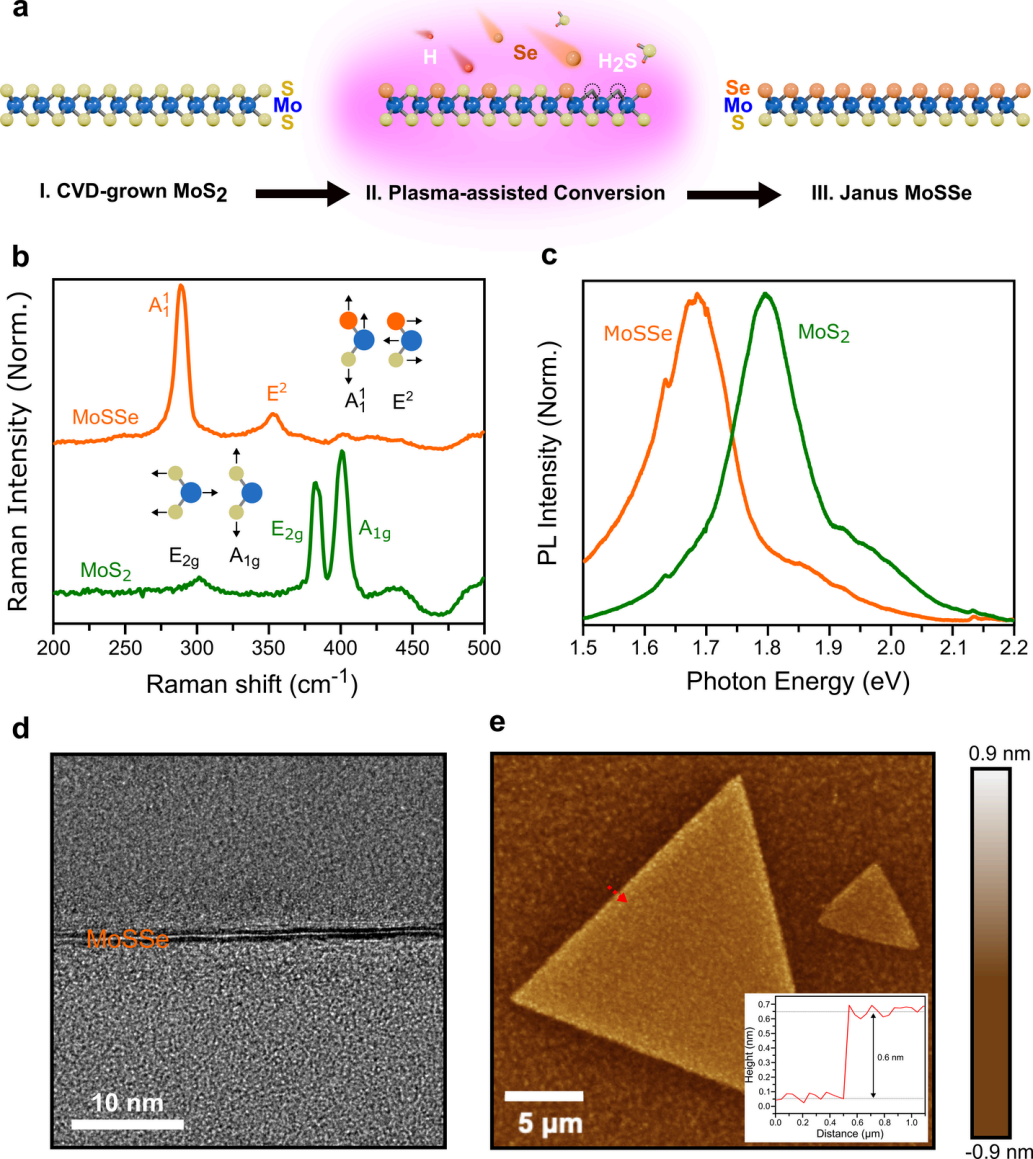
Synthesis and characterization of monolayer Janus MoSSe. (a) Schematic of the plasma-assisted conversion process from monolayer MoS_2_ to Janus MoSSe. Further details can be found in the Methods section. (b) Raman spectra of pristine MoS_2_ (green) and Janus MoSSe (orange), highlighting the characteristic shifts from the E_2g_ and A_1g_ vibrational modes of MoS_2_ to the A_1_^1^ and E^2^ vibrational modes of Janus MoSSe. (c) Photoluminescence (PL) spectra showing the emission peak redshift from MoS_2_ (green) to Janus MoSSe (orange). (d) Cross-sectional transmission electron microscopy (TEM) image confirming the monolayer structure. (e) Atomic force microscopy (AFM) topography of a typical MoSSe flake; the inset shows a height profile measured along the dotted line, indicating a monolayer thickness of ≈0.6 nm.

First, Raman and PL spectroscopy verified the structural and optical transformations into a Janus structure. The Raman spectrum of the converted material (Figure 1b) shows the characteristic out-of-plane A_1g_ and in-plane E_2g_ modes of pristine MoS_2_ (located at 401 and 383 cm^–1^) shifting markedly to A_1_^1^ and E^2^ vibrational modes^52^ of Janus MoSSe, located at 288 and 353 cm^–1^, respectively. This frequency shift, attributed to the altered chalcogen mass and broken vertical symmetry, provides clear evidence of the S–Se substitution. Complementary PL measurements (Figure 1c) reveal a corresponding redshift in the main emission peak from 1.80 eV (MoS_2_) to 1.68 eV (Janus MoSSe). Both spectral signatures are in excellent agreement with established literature values.^9,12,52^

Second, preservation of the monolayer nature after conversion was confirmed by microscopy. A cross-sectional transmission electron microscopy (TEM) image (Figure 1d) directly visualizes the single-layer structure. Furthermore, atomic force microscopy (AFM) topography (Figure 1e) shows large-area uniform flakes. A representative height profile, measured along the dotted line in Figure 1e and shown in the inset, confirms a thickness of approximately 0.6 nm, which is consistent with a monolayer of Janus MoSSe.

The atomic and electronic structure of the Janus MoSSe monolayer was investigated using STM/S. For these measurements, the synthesized MoSSe monolayers were transferred onto a substrate of graphene on a hexagonal boron nitride multilayer (h-BN)/SiO_2_, where the graphene provides a conductive backplane for tunneling, and the h-BN layer minimizes substrate roughness. Atomic-resolution STM topography images reveal a well-ordered hexagonal lattice, as shown in Figure 2a. A height profile measured across the atomic lattice (Figure 2b) yields a lattice constant of 3.25 Å, and its hexagonal symmetry is confirmed by the fast Fourier transform (FFT) of the topography (Figure 2c).

**Figure 2:**
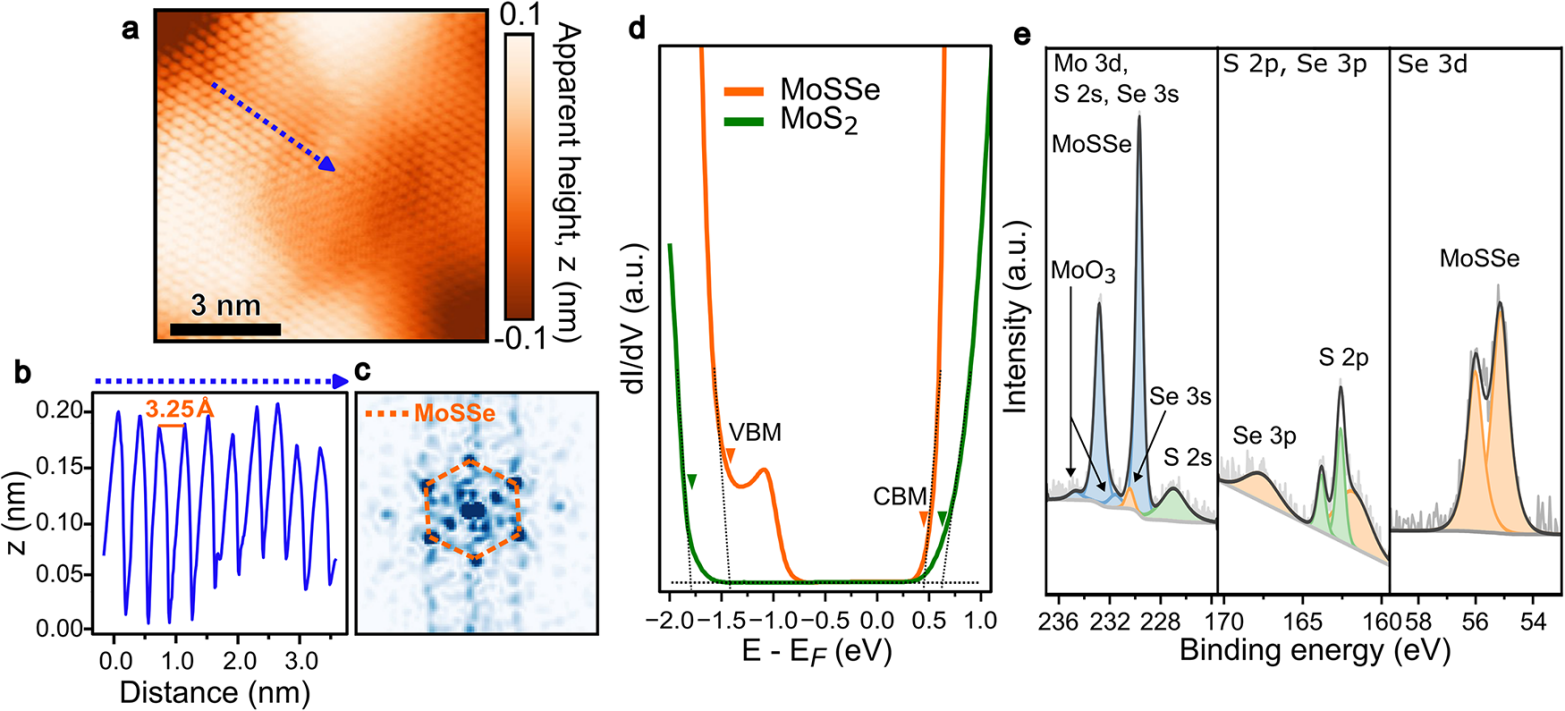
Atomic and electronic structures of monolayer Janus MoSSe. (a) Atomic-resolution STM topography images (6.5 nm × 6.5 nm) of the Janus MoSSe surface (*V*_sample_ = −1.4 V, *I*_t_ = 500 pA, *T* = 13 K). (b) Height profile along the dashed line in (a), yielding a lattice constant of 3.25 Å. (c) Fast Fourier transform (FFT) of the image in (a), confirming the hexagonal lattice symmetry. (d) Spatially averaged d*I*/d*V* spectra comparing Janus MoSSe (orange) and pristine MoS_2_ (green), revealing an upward shift of the valence band maximum (VBM) and the emergence of prominent in-gap states from −0.8 to −1.3 eV in MoSSe. The positions of the VBM and CBM, indicated by the inverted triangles, are determined from the intersection of linear fits (dashed lines) of both band edge onsets and the bandgap region. Further detail of band edge extraction can be found in previous literature.^56,57^ (e) XPS spectra of the Mo 3d/S 2s/Se 3s, S 2p/Se 3p, and Se 3d core levels, used for compositional analysis. The deconvoluted peaks for Mo (blue), S (green), and Se (orange) are shown.

To probe the electronic properties, we performed scanning tunneling spectroscopy (STS), using pristine MoS_2_ as a reference. Our STS measurements on a monolayer MoS_2_ (Figure 2d, green curve) reveal a valence band maximum (VBM) at approximately −1.76 eV and a conduction band minimum (CBM) at +0.65 eV, corresponding to a bandgap of ≈2.41 eV. This result serves as a benchmark, as it aligns with previously reported values for the monolayer MoS_2_.^53^ In comparison, the spectrum for Janus MoSSe (Figure 2d, orange curve) shows a distinct modification. While the CBM remains at a similar energy, +0.48 eV for the Janus MoSSe, the VBM is shifted upward by 0.28 to −1.48 eV. This reduces the bandgap to ≈1.96 eV, a narrowing trend that is consistent with the previous literature^54,55^ and our PL measurements (Figure 1c). The details of the impact of intrinsic dipole on STS measurement and the excitonic effect of Janus MoSSe can be found in Supplementary Note 2.

Furthermore, beyond this expected bandgap change, the Janus MoSSe spectrum exhibits a distinct plateau of in-gap states extending from −0.8 to −1.3 eV, a feature absent in pristine MoS_2_. To quantitatively characterize these states, we performed a statistical analysis of thousands of individual spectra acquired from CITS maps. The detailed statistical analysis, which can be referred to in Supplementary Note 3, reveals that these in-gap states have a mean peak position of −1.12 ± 0.09 V and an average intrinsic width (fwhm) of 0.20 V. The large standard deviation in the peak position (90 meV) provides a quantitative measure of the electronic inhomogeneity across the surface. We hypothesized that these states originate from chemical inhomogeneities introduced during the formation of the Janus MoSSe by the plasma-assisted selenization process.^9^ To test this hypothesis, we performed X-ray photoelectron spectroscopy (XPS) for compositional analysis (Figure 2e). The deconvolution of the Mo, S, and Se core-level spectra reveals an S/Se atomic ratio of approximately 1.03:0.97, close to the expectation S/Se = 1:1 for the Janus MoSSe. Given that our synthesis begins with a MoS_2_ monolayer, the presence of slight sulfur excess in the converted layer is most logically attributed to an incomplete top-layer conversion, leaving residual S atoms that act as intrinsic dopants. These sulfur dopants are thus responsible for the prominent in-gap states, fundamentally modifying the electronic structure of the Janus monolayer.

Having established that residual sulfur dopants introduce in-gap states, we next investigated their spatial distribution and local electronic impact. High-resolution STM topography (Figure 3a) reveals nanoscale regions of varying contrast, which we label as bright (Region B) and dark (Region D). This contrast is electronic in origin, as it is directly supported by the LDOS map acquired via current imaging tunneling spectroscopy (CITS) at −1.15 eV (Figure 3b).

**Figure 3:**
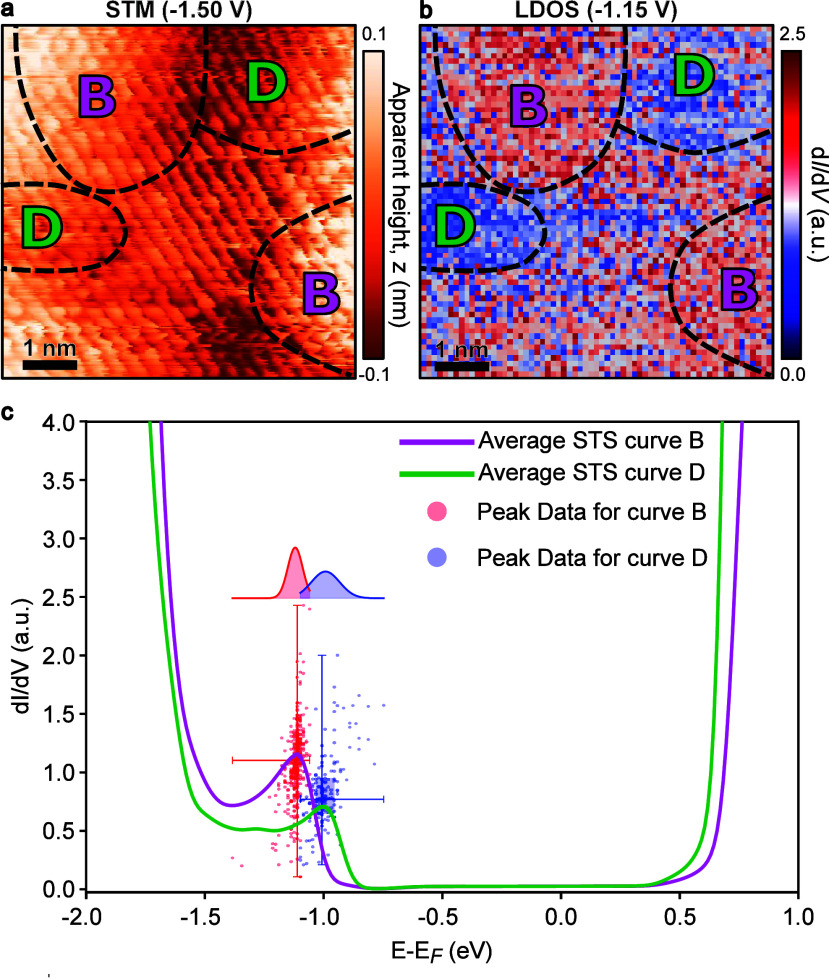
Spatially resolved electronic inhomogeneity due to sulfur dopants. (a) High-resolution STM topography showing electronically distinct bright (Region B) and dark (Region D) domains (*V*_sample_ = −1.4 V, *I*_t_ = 500 pA, *T* = 13 K). (b) Corresponding current imaging tunneling spectroscopy (CITS) map of the same area taken at −1.15 eV, confirming that the contrast originates from variations in the local density of states (LDOS). The dashed lines indicate the representative areas used for the spectral averaging and statistical analysis shown in (c). (c) Spectral analysis comparing the two regions. The main panel shows averaged STS curves for Regions B (magenta) and D (green). Superimposed are a box plot and scatter plot of individual in-gap state peak measurements (intensity vs energy), extracted from each STS curve, and their projected histograms, which quantitatively visualize the higher intensity and shallower energy level of the states in Region B.

To quantify this electronic inhomogeneity, we performed spatially resolved STS across these regions. The averaged d*I*/d*V* spectra shown in Figure 3c were extracted from the representative bright and dark areas demarcated by the dashed lines in Figure 3b. The resulting spectra show that the intensity of the in-gap state is significantly more pronounced in the bright regions. A detailed statistical analysis of hundreds of individual peak measurements in both regions provides a quantitative picture: the mean peak intensity in Region B (magenta solid line) is approximately 1.08 au, about 30% higher than 0.83 au in Region D (green solid line). Furthermore, the energetic peak position in Region B is located at a shallower mean energy (−1.12 ± 0.03 eV) with a narrower distribution compared with that in Region D (−0.99 ± 0.06 eV). Recalling our earlier finding that these residual sulfur dopants induce these in-gap states, it follows that these observed spatial variations in the in-gap states of the local electronic structure may originate from a spatial variation in the distribution of dopants. We therefore hypothesize that the bright regions (Region B), with their intense and narrowly distributed shallow in-gap states, correspond to areas of a higher local sulfur concentration. Conversely, the dark regions (Region D), with their weaker in-gap state intensity and broader state distribution, correspond to areas of lower sulfur concentration. The details of this hypothesis can be referred to Supplementary Note 4.

To validate this hypothesis, we performed density functional theory (DFT) calculations (Figure 4). The details of the DFT calculation is detailed in Supplementary Note 5. We modeled two surfaces to simulate the trend of change in electronic structure: a “sulfur-rich” surface (70% S on the top Se-layer) and a “sulfur-deficient” surface (30% S). The calculated density of states (DOS) for the sulfur-rich model exhibits prominent in-gap states near the VBM. In contrast, the sulfur-deficient model shows a weaker and broader distribution of states within the gap. This theoretical trend, that a higher sulfur concentration yields a more intense and shallower in-gap state, is consistent with our experimental observations and the resulting hypothesis. This provides compelling evidence that the bright regions (B) are sulfur-rich, while the dark regions (D) are sulfur-deficient. We acknowledge that this study focuses on the electronic signature of these dopants rather than their single-atom chemical identification. Future work combining our STM/STS with advanced, low-dose analytical techniques (e.g., ADF-STEM, EDS/EELS) would be invaluable for providing a supportive correlation between the atomic structure, chemical identity, and the electronic properties we have characterized.

**Figure 4:**
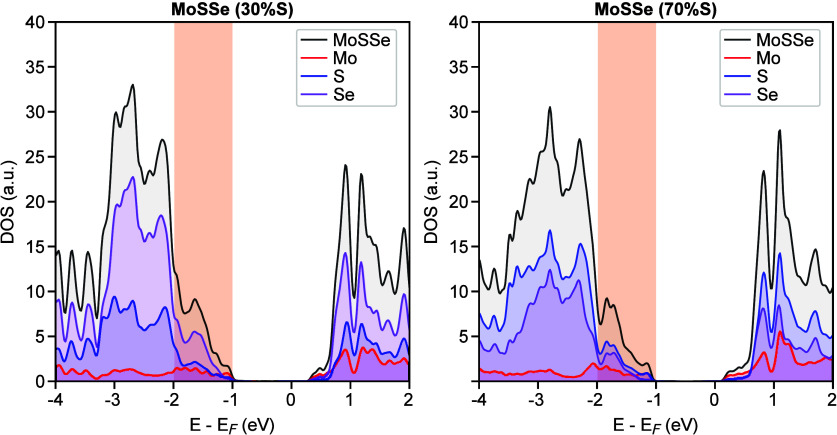
DFT-calculated density of states for MoSSe with varying sulfur concentration. Calculated total density of states (black) is shown overlaid with the projected density of states (PDOS) for Mo (red), S (blue), and Se (purple) atoms. Two models are compared. Left panel: a “sulfur-deficient” surface with 30% S concentration on the top Se-layer (MoSSe (30% S)), and right panel: a “sulfur-rich” surface with 70% S concentration on the top Se-layer (MoSSe (70% S)). The PDOS contribution from Mo atoms has been scaled to account for the tunneling distance in STS measurements, allowing for a more direct comparison with experimental spectra (see Supplementary Note 5 for details). The calculations demonstrate that a higher sulfur concentration leads to a more prominent in-gap state feature near the valence band maximum. The orange shaded regions highlight this area of the in-gap states.

Beyond the compositional inhomogeneity from intrinsic sulfur dopants, we identified another class of native imperfections in Janus MoSSe: bias-dependent charge defects. These defects are strikingly revealed through the STM topography. While the surface appears relatively clean at positive sample bias (Figure 5a, + 1.5 V), distinct, bright, atom-sized features emerge across the same area when switching to negative bias (Figure 5b, −1.5 V). This bias-dependent appearance is direct evidence that these features are electronic in nature rather than topographic structures such as particles or clusters.

**Figure 5:**
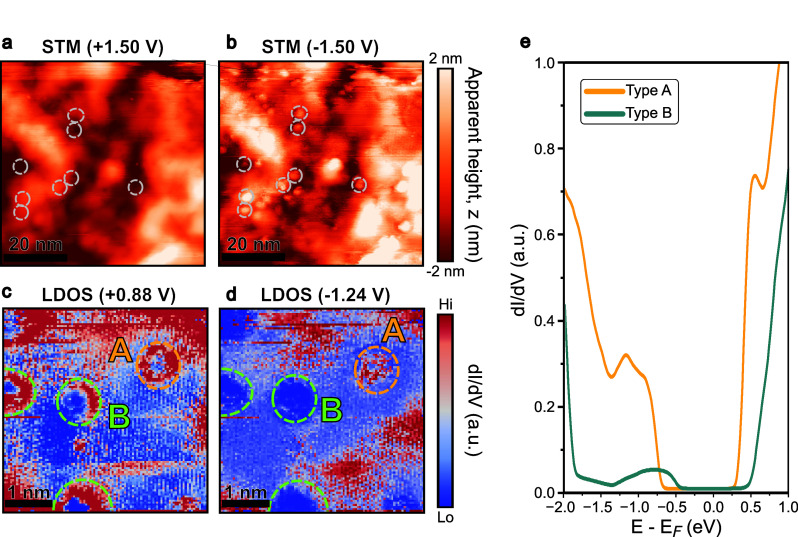
Bias-dependent imaging and electronic signatures of native charge defects. (a, b) STM topography of the same region imaged at (a) positive (+1.5 V) and (b) negative (−1.5 V) sample bias, revealing the emergence of bright, localized charge defects at negative bias. (c, d) CITS maps of a selected area with multiple defects at (c) +0.88 V (conduction band states) and (d) −1.24 V (in-gap states), *V*_sample_ = −0.8 V, *I*_t_ = 300 pA, and *T* = 13 K. The distinct appearance at negative bias allows for the classification of defects into Type A (bright protrusion) and Type B (dark depression). (e) Averaged d*I*/d*V* spectra, extracted from the defect areas demarcated in (c) and (d), compare the distinct electronic fingerprints of a Type A (yellow curve) and a Type B (green curve) defect.

Having established their electronic origin, we performed high-resolution CITS mapping at an atomic-scale spatial resolution to investigate the spatial characteristics of these defects (see Video S1 for complete electronic structure evolution in real space). Intriguingly, the LDOS maps reveal that these defects are not all identical. While all defects exhibit a bright ring-like structure in the conduction band states (Figure 5c, +0.88 V), their appearance diverges in the in-gap states (Figure 5d, −1.24 V). Based on this, we classify them into two categories: Type A, which remains as a bright protrusion, and Type B, which appears as a dark depression in negative bias.

This classification is validated by their distinct electronic fingerprints, as shown in Figure 5e, which displays the STS spectra averaged from within the defect areas demarcated by dashed circles in Figure 5c,d. The Type A defect is characterized by a high-intensity continuum of in-gap states from −0.7 to −1.3 eV, which merges with the valence band edge. This, combined with a downward shift of the CBM, collectively shrinks the local effective bandgap. In contrast, the spectrum for the Type B defect exhibits only a weak and broadly distributed band of in-gap states, alongside a markedly lower overall intensity of its LDOS.

While the spatially averaged spectra confirm the distinct electronic nature of Type A and B defects, the ring-like features observed in CITS maps (Figure 5c) strongly suggest that the electronic structure is also highly inhomogeneous within a single defect type. To resolve these intradefect variations, we performed high-resolution line-profile STS at a high spatial resolution of 1.3 Å across individual defects, with the measurement trajectories, as shown in Figure 6a,b. The resulting spatially resolved spectra, presented as waterfall plots of representative curves in Figure 6c,d, reveal the internal electronic structure distribution of each defect type.

**Figure 6:**
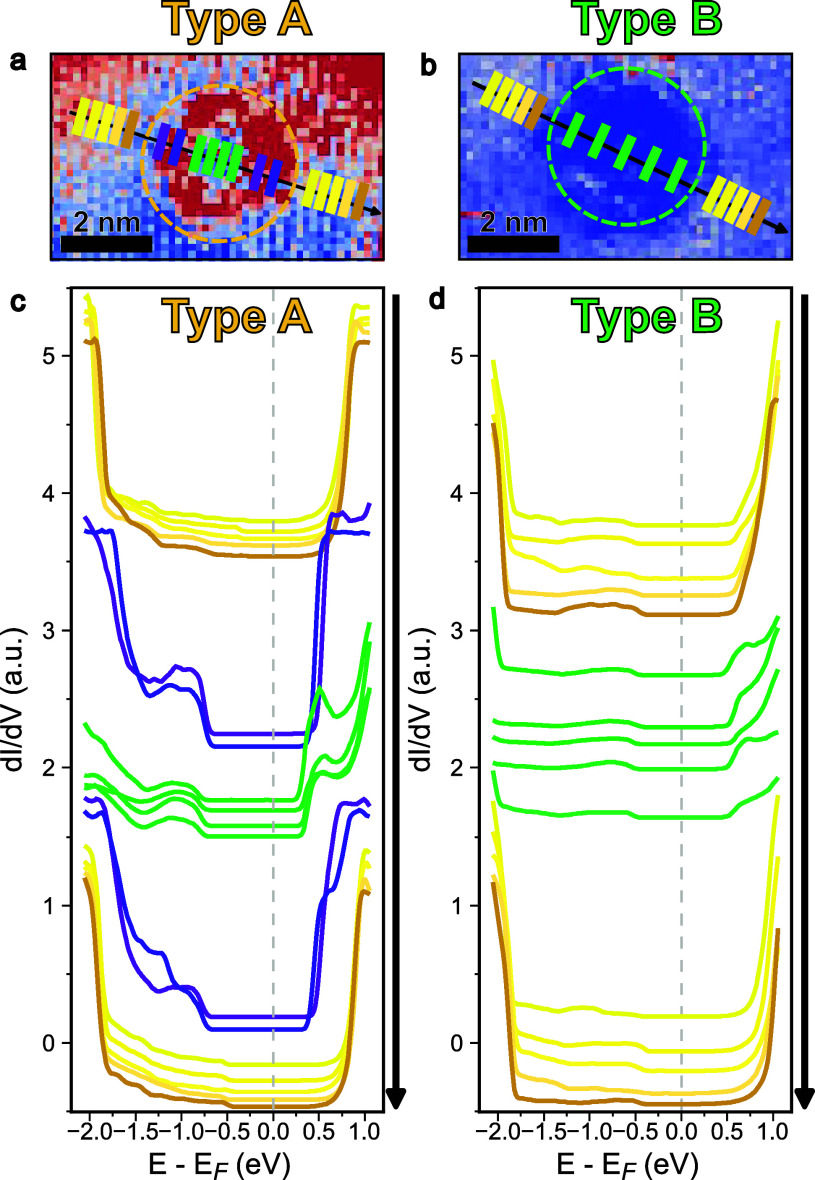
Spatially resolved spectroscopy of individual charge defects. (a, b) CITS maps of a representative Type A and Type B defect, selected from the overview maps in Figure 5c,d at +0.88 and −1.24 V, respectively. The colored markers indicate the trajectory of the line-profile STS measurements. (c, d) Waterfall plots showing representative STS spectra extracted from different regions along the line profiles in (a) and (b), respectively. The spectra are color-coded to represent the surrounding normal region (yellow), the defect shell (purple for Type A), and the defect core (green). These plots provide an excellent way to visualize the evolution of the LDOS across the (c) Type A and (d) Type B defects.

For the Type A defect (Figure 6c), a clear core–shell electronic structure across the defect is observed. The spectra from the “shell” region (purple curves) exhibit the most intense in-gap states, located between −0.7 and −1.3 eV. In the “core” (green curves), however, the intensity of these in-gap states is weaker, and the overall LDOS of both valence and conduction bands is reduced compared to the shell and the surrounding normal regions (yellow curves). This spatially dependent intensity modulation explains the core–shell contrast observed in the CITS maps (Figure 5c).

In stark contrast, the Type B defect (Figure 6d) lacks this core–shell structure. The primary characteristic of Type B is a relatively uniform reduction in the intensity of the LDOS across the entire defect area compared to that of the normal region. Although weak in-gap states are present, this overall lower electronic state density characterizes the defect as a less-conductive, insulating scattering center.

While the representative spectra in Figure 6 reveal the core electronic features, the position-dependent d*I*/d*V* maps (Figure 7), constructed from the high-resolution CITS data, provide a full, continuous visualization of the local band alignment across each defect. The Type A defect (Figure 7a) creates a deep potential well, shrinking its effective bandgap, defined as the energy separation between the CBM and the in-gap state, by nearly 50%, from 2.0 eV for the normal region to 1.04 eV for the core of the Type A defect. This is caused by a substantial downward shift of the CBM (≈0.45 eV) and an upward lift of the in-gap states (≈0.51 eV) in the core of the Type A defect. Critically, while the bandgap is narrowest at the defect core, the LDOS intensity is weakest there and strongest in the surrounding shell. This confirms that the Type A defect functions as a resonant cavity-like conductive charge trap, where carriers are efficiently captured and accumulate at the periphery.

**Figure 7:**
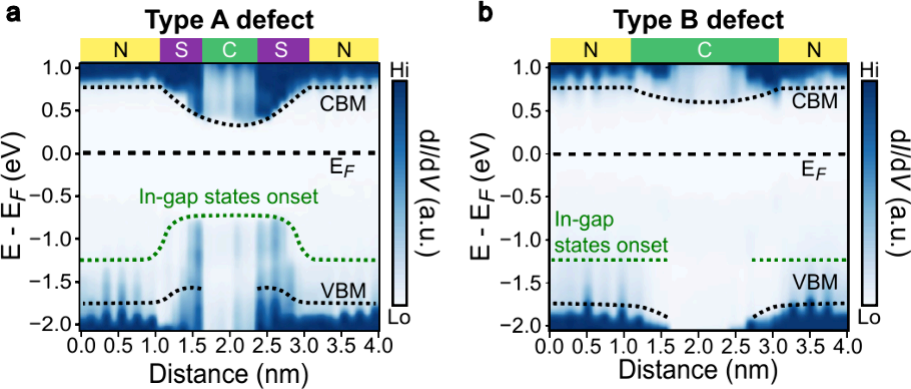
Electronic band alignment across individual charge defects. (a, b) Position-dependent d*I*/d*V* maps constructed from the complete line-profile STS data set for a (a) Type A and (b) Type B defect. The map for Type A reveals a deep potential well, where the highest intensity of the in-gap states (the onset of in-gap states is indicated by a green dashed line) is localized in a conductive shell surrounding a weaker core. In contrast, the map for Type B can be characterized by a uniform reduction of state density and a distinct downward bending of the VBM. The letters C, S, and N on the bars at the top denote the core, shell, and normal regions, respectively. The black dashed lines are visual guides for the VBM, CBM, and Fermi level (*E*_F_).

In contrast, the band alignment of the Type B defect (Figure 7b) is distinguished by an opposite bending of the valence band. While its CBM bends slightly inward by ≈0.17 eV, its VBM shifts downward, away from the Fermi level. This unique band profile, combined with a uniformly lower LDOS intensity compared to the surrounding normal region, characterizes the Type B defect as an insulating scattering center that would impede local charge transport.

While our STM/S analysis provides a clear electronic classification, it does not directly resolve the chemical identity of these atomic-scale defects. However, based on the literature^51,54^ and our DFT simulations (Figure S7), the significant bandgap reduction emerges with the presence of selenium vacancies (V_Se_). This theoretical result may be in accord with our experimental observations of the Type A defect, suggesting that selenium vacancies are a likely origin for these charge-trapping centers. The origin of the Type B scattering centers with their broader and less-defined features may be related to local structural disorder induced by plasma bombardment during the Janus conversion process. The details of the Type A and Type B defect attribution can be found in Supplementary Note 6.

Collectively, these findings demonstrate that the electronic structure of synthesized Janus MoSSe is a complex landscape populated by a hierarchy of imperfections. Based on the statistical analysis of all large-area STM images with charge defects (details of statistical analysis can be referred to Supplementary Note 8), we determined that the Type A and B defects have a combined density of (5.05 ± 2.36) × 10^11^ cm^–2^. We suppose that this nonuniform distribution of dopants and functionally distinct defects may be a primary reason for the discrepancy between the theoretically predicted superiority of Janus MoSSe and its actual, often limited, device performance.^9,12,58^ While these imperfections can be detrimental, they could also be harnessed: the inhomogeneous sulfur dopant and the Type A defect may serve as active centers for catalysis^42,43^ and sensing,^45^ and the charge-trapping nature of the Type A defect is the basis for neuromorphic devices,^48,50^ whereas the insulating sites (Type B) would likely impede carrier transport in all such applications. This study pinpoints that a potential path forward lies in defect engineering. By careful tuning of the synthesis process, it may be possible to control the formation of these defects, thereby mitigating their detrimental effects and ultimately improving the performance of Janus-based electronic devices.

## Conclusions

In conclusion, our comprehensive atomic-scale STM/S investigation has mapped the complex electronic structure of the synthesized monolayer Janus MoSSe, revealing that it is dominated by a variety of native imperfections. We provide direct evidence that residual sulfur dopants, as a consequence of incomplete synthesis, create a spatially inhomogeneous electronic structure. These dopants induce a broad band of in-gap states at around −1.1 eV, which are demonstrably more intense and energetically 0.13 eV shallower in sulfur-rich regions.

Furthermore, we discovered and characterized two functionally distinct native charge defects with a characteristic size of ≈2.5 nm. The Type A defect functions as a resonant cavity-like conductive charge trap, forming a deep potential well that reduces the local effective bandgap by nearly 50%. In contrast, the Type B defect acts as an insulating scattering center, characterized by a markedly lower LDOS compared with the surrounding material and a downward bending of its valence band. The coexistence of these defect types, one capturing the carrier and the other impeding their flow, would therefore disrupt carrier transport in an electronic device.

The characterization of this imperfection provides microscopic insight into the frequently observed performance gap in Janus MoSSe devices. Based on our combined experimental and theoretical results, we propose the origins of these defects. The electronic signature of the Type A charge trap is consistent with a selenium vacancy (V_Se_), as suggested by our DFT calculations. The Type B scattering center, with its lower DOS and broader spectral features, is likely associated with a local structural disorder induced by the plasma synthesis process. This work underscores that a primary bottleneck for achieving superior performance lies in controlling these synthesis-induced imperfections, pointing toward defect engineering as a promising path forward to unlock the full potential of Janus materials for future electronic devices.

## Experimental Section

### Chemical Vapor Deposition (CVD) of Monolayer MoS_2_

Monolayer MoS_2_ flakes were synthesized by the CVD method. Briefly, in a quartz boat placed in the center of the furnace, a mixture of MoO_3_ (3 mg, 99.5% purity, Alfa Aesar) and NaCl (0.5 mg, 99.5% purity, Showa Chemical Co.) was placed 3 cm away from the sapphire substrates. S powder (1 g, 99.5–100.5% purity, Sigma-Aldrich) was placed in a crucible located upstream of the furnace with an annealing temperature of 200 °C. The MoS_2_ growth was carried out at 830 °C for 5 min under an atmospheric pressure with high-purity Ar (200 sccm) as the carrier gas. After growth, the system was cooled naturally to room temperature.

### Janus Conversion Process

The as-grown MoS_2_ was transferred onto a SiO_2_/Si substrate by using a standard wet transfer method. The MoS_2_/SiO_2_ sample was then placed in a plasma-assisted selenization furnace to convert the top sulfur layer into selenium, forming Janus MoSSe, as illustrated in Figure 1. The base pressure was pumped down below 1 × 10^–2^ Torr before starting the selenization process to prevent contamination and impurity. During selenization, the furnace temperature was ramped up to 200 °C at a rate of 10 °C/min and held for 45 min. Throughout the process, hydrogen gas (H_2_) was continuously flowed at 250 sccm. Once the target temperature was reached, a 20 W plasma was ignited for 5 min to generate hydrogen (H_2_) radicals within the chamber. These H_2_ radicals reacted with the top-layer sulfur atoms in MoS_2_, forming H_2_S and inducing sulfur vacancies. Subsequently, selenium atoms filled these vacancies, resulting in the formation of Janus MoSSe. After the sample was cooled to room temperature, the successful synthesis of Janus MoSSe on the SiO_2_ substrate was confirmed. For STM measurement, Janus MoSSe flakes were transferred to the target substrate by the wet transfer method. Further details can be found in previous literature.^9^

### STM Measurement Setup

The STM measurements were conducted in the commercial ultrahigh vacuum STM system (Infinity SPM Lab, Scienta Omicron) with a base pressure of 6 × 10^–11^ mbar. All STM images and STS spectra were acquired at 13K temperature in the constant-current mode by using electrochemical etching tungsten tip with a sample bias configuration. The d*I*/d*V* spectra are acquired using a lock-in amplifier with a modulation amplitude of 10 mV and frequency of 973 Hz. Each spectrum was recorded over a bias range from +1.05 to −2.05 V, consisting of 401 data points with a total acquisition time of 4 s per curve. The Fermi level (*E*_F_) is defined at zero sample bias, where the Fermi levels of the tip and sample are aligned.

### Materials Characterization

XPS measurements were performed using a commercial XPS system (ULVAC-PHI, PHI 5000 VersaProbe III) equipped with a monochromatic Al Kα radiation source (1486.6 eV). To eliminate charging effects that could distort peak shapes and compromise quantitative accuracy, an argon ion (Ar^+^) charge neutralization gun was employed during all of the measurements. For quantitative elemental analysis, the spectra were fitted using a mixed Gaussian–Lorentzian function with a 70:30 ratio (GL(30)). A LABRAM HR 800 UV Raman spectrometer equipped with a 532 nm laser was used for Raman spectroscopy measurement. The LABRAM HR 800 UV system was used to conduct the PL spectroscopy characterization. The TEM specimen was prepared by the focused ion beam (FIB) technique and examined using a JEOL JEM-F200 microscope.

## Supplementary Material





## Data Availability

The data that support the findings of this study are available from the corresponding author upon reasonable request.

## Notes

Janus synthesis detail; impact of intrinsic dipole on STS and excitonic effect; statistical analysis on in-gap states of Janus MoSSe; origin of in-gap states in Janus MoSSe; computational detail; defect types attribution; statistical analysis of charge defect (PDF)
Movie of complete evolution of MoSSe electronic structure in real space (MP4)
