# Supplementary material for: Atomically Resolved Defects Modulate Electronic Structure in Plasma-Assisted 2D Janus MoSSe Monolayers
Source: ACS Nano. 2025 Dec 9;19(50):42365–74. doi: 10.1021/acsnano.5c14446 (PMC12752698; doi:10.1021/acsnano.5c14446)
Supplement: Supplementary file 1 [file nn5c14446_si_001.pdf]

Atomically-Resolved Defects Modulate Electronic Structure in Plasma-Assisted 2D Janus MoSSe Monolayers

Zi-Liang Yang[1,2,3,6], Yu-Chieh Lin[1], Mayur Chaudhary[4], Li-Sheng Lin[1], Chih-Yang Huang[3,5,6], You-Jie Lin[4], Jyh-Pin Chou[*1], Li-Chyong Chen[2,3,5], Kuei-Hsien Chen[5,6], Yu-Lun Chueh[*4,7,8,9], Ya-Ping Chiu[*1,2,3,6]

[1]Graduate School of Advanced Technology, National Taiwan University, Taipei 10617, Taiwan

[2]Department of Physics, National Taiwan University, Taipei 10617, Taiwan

[3]Center of Atomic Initiative for New Materials, National Taiwan University, Taipei 10617, Taiwan

[4]Department of Materials Science and Engineering, National Tsing-Hua University, Hsinchu 30013, Taiwan

[5]Center for Condensed Matter Sciences, National Taiwan University, Taipei City 10617, Taiwan

[6]Institute of Atomic and Molecular Sciences, Academia Sinica, Taipei 10617, Taiwan

[7]College of Semiconductor Research, National Tsing-Hua University, Hsinchu 30013, Taiwan

[8]Department of Physics, National Sun Yat-sen University, Kaohsiung 80424, Taiwan

[9]Department of Materials Science and Engineering, Korea University, Seoul, 02841, Republic of Korea

[*]Corresponding author. E-mail: ypchiu66@ntu.edu.tw (Y.-P.C.), jpchou@ntu.edu.tw (J.-P.C.) & ylchueh@mx.nthu.edu.tw (Y.-L.C)

Supplementary Note 1

Janus synthesis detail

The conversion of MoS_2 to MoSSe is primarily dictated by temperature and plasma conditions. At low temperature (200 °C), selective top-layer S-to-Se substitution produces an ordered Janus MoSSe phase with relatively few defects. As the temperature rises (400 °C), enhanced chalcogen diffusion results in a Janus + alloy state, where partial S/Se intermixing increases substitutional disorder. At high temperature (600 °C), complete interdiffusion yields a fully alloyed lattice, accompanied by significant structural disorder and chalcogen vacancies.

While temperature governs the overall phase progression (Janus → Janus+alloy → alloy) as shown in **Figure S1**, the plasma environment controls the defects by governing sulfur defects, which are then replaced by selenium. Higher plasma power accelerates S desorption and Se incorporation but also increases the likelihood of defect formation, including vacancies, antisites, and local amorphization. Thus, a careful balance between temperature and plasma power is essential to achieve a stable Janus MoSSe phase with minimized defects.

Figure S2 shows the Raman spectra for Janus-MoSSe under different plasma time and plasma power. At 200 °C, plasma exposure time governs the conversion of MoS_2 to Janus MoSSe, with longer durations leading to clear Janus Raman modes. Plasma power, however, mainly controls the defect density: moderate power (~20 W) enables clean Janus formation, while higher power broadens the Raman peaks, indicating disorder and defect generation. Thus, temperature drives the conversion, while plasma power regulates defect formation.

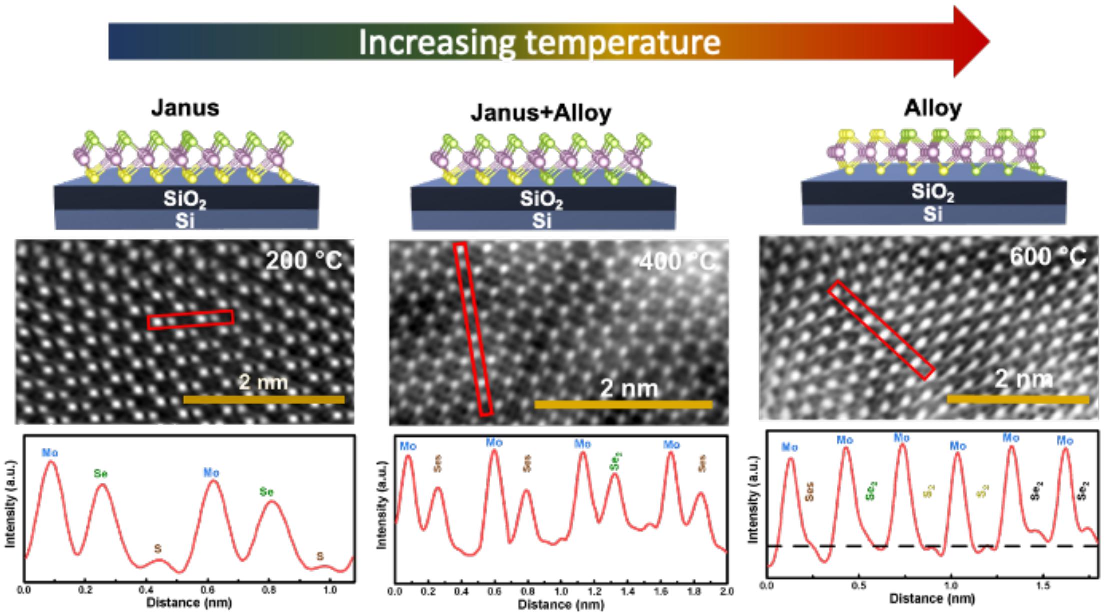

Figure S1 | Different structure formation at different temperatures.

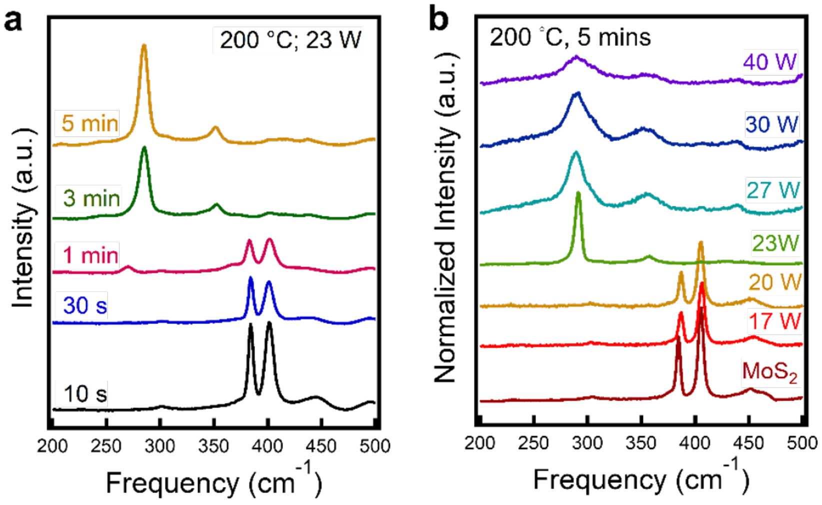

Figure S2 | Raman spectra for the formation of Janus-MoSSe under: (a) different plasma time and (b) different plasma powers

We have also summarized the key parameters used in this manuscript to synthesize Janus-MoSSe in the supplementary **Table S1**.

Table S1 | Parameters for the formation of Janus-MoSSe monolayer.

Substrate temperature (^{o}C)	Plasma power (W)	Gas ratio (H_2/N_2) in sccm	Plasma time (min)
200	23	60/30	5

Reproducibility data in the form of representative Raman spectra from different synthesis batches and from different positions on the same wafer, confirming the uniformity of our method (see **Figures S3** and **S4**).

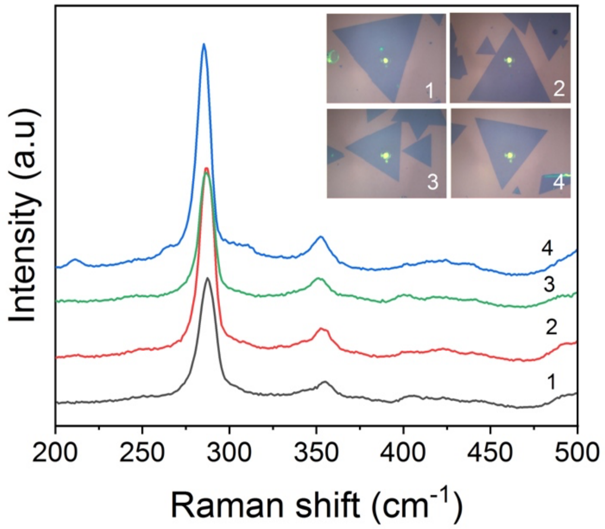

Figure S3 | Raman spectra collected from four different batch of samples.

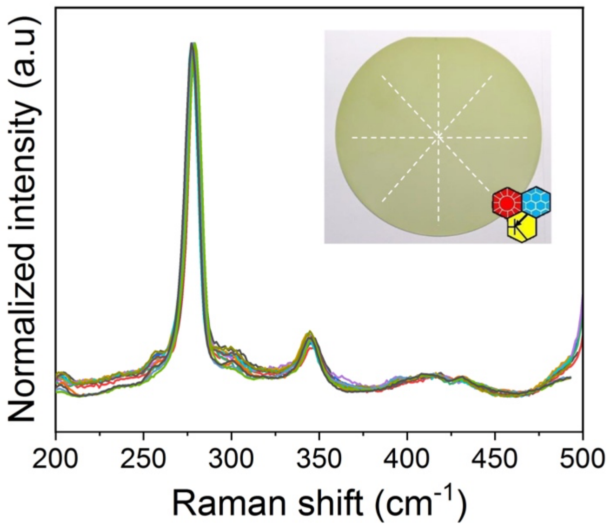

Figure S4 | Raman spectra collected from one batch of sample. The spectra were obtained at 13 different positions across the substrate.

Supplementary Note 2

Impact of intrinsic dipole on STS and excitonic effect

1. On the influence of the out-of-plane dipole on STS band edge determination:

The intrinsic out-of-plane dipole in Janus MoSSe indeed leads to different surface potentials on the S- and Se-terminated surfaces. As confirmed by Kelvin Probe Force Microscopy (KPFM) studies in the literature, this potential difference can be around 100 meV[1]. In principle, such a difference could lead to a measurement-dependent offset in the STS spectra[2].

As shown in **Figure 3** in the main text, Our Janus MoSSe surface, formed by incomplete selenization of MoS_2, contains nanoscale regions with varying concentrations of residual sulfur dopants. We can therefore consider S-deficient regions (Region D in our manuscript) as having a stronger local Janus character and thus a stronger effective dipole, while S-rich regions (Region B) are with a weaker effective dipole.

We observed a clear and systematic shift of band edges between the averaged spectra from the S-rich (B) and S-deficient (D) areas in **Figure 3c**. The spectrum from the S-deficient region—which we propose has the stronger local dipole and thus a lower CB band edge position—is shifted entirely to more negative energies. This shift is consistent with the expected influence of a stronger out-of-plane dipole causing downward band bending and making the sample appear more n-type.

Therefore, not only do we acknowledge the dipole's influence, but we also show that this effect is observable in our data and provides a self-consistent explanation for the electronic inhomogeneity on the MoSSe surface.

2. On the excitonic effect in the Janus system:

By combining our experimental data, we calculated the exciton binding energy (E_{bind}) using the relation $E_{bind} = E_{qp} - E_{opt}$, where the quasiparticle bandgap (E_{qp}) is determined from STS and the optical bandgap (E_{opt}) from photoluminescence (PL)[3].

Our analysis yields a significant finding: the exciton binding energy for our Janus MoSSe is **0.28 eV** (E_{qp} = 1.96 eV; E_{opt} = 1.68 eV), which is less than half of that for the pristine MoS_2 monolayer measured under identical conditions (**0.61 eV**). This pronounced reduction in E_{bind} for Janus MoSSe is consistent with theoretical predictions, which attribute this phenomenon to the intrinsic dipole of the Janus structure[4]. The built-in electric field spatially separates the electron and hole combination, thus weakening their mutual Coulombic attraction and lowering the energy required to dissociate the exciton.

We note that our experimentally determined E_{bind} of 0.28 eV for MoSSe is lower than some theoretical values for a freestanding monolayer (E_{bind} = 0.72 eV)[5-7]. This phenomenon is also consistent with the reported literatures[8, 9] that the **dielectric screening** from the underlying conductive substrate weakens the Coulomb interaction, resulting lower exciton binding energy.

The implications of this experimental finding are important. A significantly lower exciton binding energy facilitates the dissociation of photogenerated excitons into free carriers, which is a critical step for efficient photocurrent generation. This result, therefore, provides strong experimental evidence that Janus MoSSe is an excellent candidate for light-harvesting applications such as photovoltaics, photocatalysis, and photodetectors, precisely because its unique structure is inherently designed to promote charge separation.

References:

(1) Guo, Y.; Lin, Y.; Xie, K.; Yuan, B.; Zhu, J.; Shen, P.-C.; Lu, A.-Y.; Su, C.; Shi, E.; Zhang, K.; et al. Designing artificial two-dimensional landscapes via atomic-layer substitution. *Proceedings of the National Academy of Sciences* **2021**, *118* (32), e2106124118. DOI: 10.1073/pnas.2106124118.

(2) Feenstra, R. M. Electrostatic potential for a hyperbolic probe tip near a semiconductor. *Journal. of Vacuum Science & Technology B: Microelectronics and Nanometer Structures* **2003**, *21* (5), 2080. DOI: 10.1116/1.1606466.

(3) Qiu, Z.; Trushin, M.; Fang, H.; Verzhbitskiy, I.; Gao, S.; Laksono, E.; Yang, M.; Lyu, P.; Li, J.; Su, J.; et al. Giant gate-tunable bandgap renormalization and excitonic effects in a 2D semiconductor. *Sci. Adv.* **2019**, *5* (7), eaaw2347. DOI: 10.1126/sciadv.aaw2347.

(4) Mehdipour, H.; Kratzer, P. Structural defects in a Janus MoSSe monolayer: A density functional theory study. *Physical Review B* **2022**, *106* (23). DOI: 10.1103/physrevb.106.235414.

(5) Hu, B.; Qian, T.-X.; Zhou, J.; Ding, Y.; Cai, T.; Ju, S. Quasiparticle Band Structure, Exciton, and Optical Property in Janus Structures of Transition-Metal Dichalcogenide Monolayers. *ACS Omega* **2025**, *10* (28), 30924–30934. DOI: 10.1021/acsomega.5c03536.

(6) Li, F.; Wei, W.; Huang, B.; Dai, Y. Excited-State Properties of Janus Transition-Metal. Dichalcogenides. *The Journal of Physical Chemistry C* **2020**, *124* (2), 1667–1673. DOI: 10.1021/acs.jpcc.9b09097.

(7) Long, C.; Dai, Y.; Jin, H. Effect of point defects on electronic and excitonic properties in Janus-MoSSe monolayer. *Physical Review B* **2021**, *104* (12). DOI: 10.1103/physrevb.104.125306.

(8) Raja, A.; Chaves, A.; Yu, J.; Arefe, G.; Hill, H. M.; Rigosi, A. F.; Berkelbach, T. C.; Nagler, P.; Schüller, C.; Korn, T.; et al. Coulomb engineering of the bandgap and excitons in two-dimensional materials. *Nature Communications* **2017**, *8* (1), 15251. DOI: 10.1038/ncomms15251.

(9) Itzhak, R.; Suleymanov, N.; Minkovich, B.; Kartvelishvili, L.; Kostianovski, V.; Korobko, R.; Hayat, A.; Goykhman, I. Exciton Manipulation via Dielectric Environment Engineering in 2D Semiconductors. *ACS Applied Optical Materials* **2025**, *3* (6), 1330–1338. DOI: 10.1021/acsaom.5c00105.

Supplementary Note 3

Statistical analysis on in-gap states of Janus MoSSe

We performed a detailed statistical analysis on over 10,000 individual spectra from our CITS data, as shown in **Figure S5**. After a robust baseline subtraction using an Asymmetric Least Squares (ALS) algorithm, the in-gap state feature was fitted with Gaussian function to extract its peak position and Full Width at Half Maximum (FWHM).

The analysis yields a mean peak position of -1.12 ± 0.09 eV and a mean FWHM of 0.20 ± 0.06 eV.

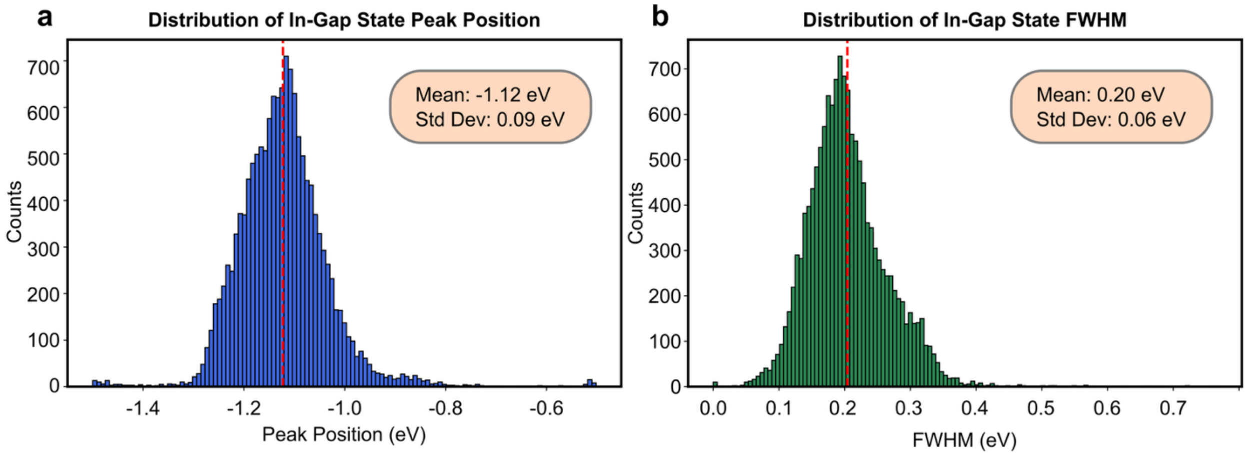

Figure S5 | Statistical analysis on the in-gap state of Janus MoSSe.

Analysis was performed on 14,221 individual d*I*/d*V* spectra extracted from CITS maps of the Janus MoSSe surface. A baseline was subtracted from each spectrum using an Asymmetric Least Squares (ALS) algorithm, and the resulting in-gap state feature was fitted with a Gaussian profile to determine its parameters. **(a)** The distribution of the in-gap state peak positions is centered at a mean energy of -1.12 eV with a standard deviation of 0.09 eV. This standard deviation provides a quantitative measure of the spatial electronic inhomogeneity across the surface. **(b)** The distribution of the Full Width at Half Maximum (FWHM) of the states reveals a mean intrinsic width of 0.20 eV with a standard deviation of 0.06 eV.

Supplementary Note 4

Origin of in-gap states in Janus MoSSe

The in-gap states do not arise from simply reproducing a MoS_2-like band alignment. Instead, they are localized defect states, and our hypothesis is based on a process of elimination and validation:

- **Experimental Constraints (Process of Elimination):** (1) First, we considered the possible origins of these states based on our experimental context. Our synthesis process starts with MoS_2 monolayer and involves the substitution of top-layer S with Se, with no intentional modification to the Mo lattice. (2) Furthermore, STM studies on TMDs have well-established that Mo-related defects (e.g., vacancies, substitutions) typically exhibit distinct and recognizable topographic signatures[1-3]. In our STM topography images, we do not observe such Mo-defect signatures in the regions where the in-gap states are prevalent.
- **Hypothesis Formulation:** Concurrently, our XPS analysis confirms the presence of unreplaced sulfur atoms on the top layer of Janus MoSSe. Therefore, the most logical and self-consistent hypothesis is that the observed in-gap features are electronic defect states arising from these residual sulfur atoms acting as isoelectronic substitutional defects within the top Se-layer.
- **Hypothesis Validation with DFT:** To test this hypothesis, we performed the DFT calculations (now **Figure 4** and **Figure S6**, details in **Supplementary Note 5**). The results provide validation for our hypothesis. The PDOS analysis shows that as the concentration of substitutional S atoms on the top surface increases (from 30% to 70%), there is a corresponding and significant increase in the density of states located just above the VBM. Crucially, the PDOS projection reveals that these emergent in-gap states are dominated by S p-orbital character, directly linking them to the presence and concentration of the sulfur

dopants. This occurs because a substitutional S atom locally perturbs the Janus structure, leading to a unique hybridization with neighboring Mo d-orbitals that creates these localized shallow states.

Given that STM/STS is an inherently surface-sensitive technique, it is physically reasonable that our measurements are strongly influenced by electronic structure changes in the top atomic layer. In summary, our assignment is based on a convergence of evidence from our synthesis method, STM topography (absence of Mo defects), XPS analysis (presence of residual S), STS measurements (presence of in-gap states), and is ultimately validated by our DFT calculations (S-character of these states).

References:

(1) Trainer, D. J.; Nieminen, J.; Bobba, F.; Wang, B.; Xi, X.; Bansil, A.; Iavarone, M. Visualization of defect induced in-gap states in monolayer MoS2. *npj 2D Materials and Applications* **2022**, *6* (1). DOI: 10.1038/s41699-022-00286-9.

(2) Kozhakhmetov, A.; Stolz, S.; Tan, A. M. Z.; Pendurthi, R.; Bachu, S.; Turker, F.; Alem, N.; Kachian, J.; Das, S.; Hennig, R. G.; et al. Controllable p-Type Doping of 2D WSe2 via Vanadium Substitution. *Advanced Functional Materials* **2021**, 2105252. DOI: 10.1002/adfm.202105252 (accessed 2021-09-02T17:24:42).

(3) Schuler, B.; Lee, J. H.; Kastl, C.; Cochrane, K. A.; Chen, C. T.; Refaely-Abramson, S.; Yuan, S.; van Veen, E.; Roldan, R.; Borys, N. J.; et al. How Substitutional Point Defects in Two-Dimensional WS_2 Induce Charge Localization, Spin-Orbit Splitting, and Strain. *ACS Nano* **2019**, *13* (9), 10520–10534. DOI: 10.1021/acsnano.9b04611.

Supplementary Note 5

Computational detail

First-principles density functional theory (DFT) calculations were performed using the Vienna *Ab initio* Simulation Package (VASP)[1], employing a plane-wave basis set along with the projector augmented-wave (PAW)[2] method. The electronic structure calculations were conducted with an energy cutoff of 400 eV. For the unit cell of MoSSe, 6 × 6 × 1 Monkhorst–Pack *k*-point mesh was used for structural relaxation, while a denser 12 × 12 × 1 *k*-point was adopted for density of states (DOS) calculations to ensure convergence. Exchange–correlation interactions were treated using the generalized gradient approximation (GGA) in the form of the Perdew–Burke–Ernzerhof (PBE)[3] functional. To accurately describe the weak van der Waals interactions between the monolayer MoSSe and the graphene substrate, the nonlocal vdW-DF functionals (PBE+rVV10L)[4] was introduced. The convergence criterion for the self-consistent field calculation was set to 1×10^{-6} eV, and the structural optimization was carried out until the Hellmann–Feynman forces on each atom were less than 0.01 eV/Å.

The optimized lattice constants for MoSSe and graphene were found to be 3.25 Å and 2.47 Å, respectively, which are consistent with previously reports[5, 6]. A $\sqrt{37} \times \sqrt{37}$ supercell of MoSSe and an 8 × 8 supercell of graphene were employed, resulting in a lattice mismatch of less than 0.1%, ensuring structural stability during the ionic relaxation steps in the calculations.

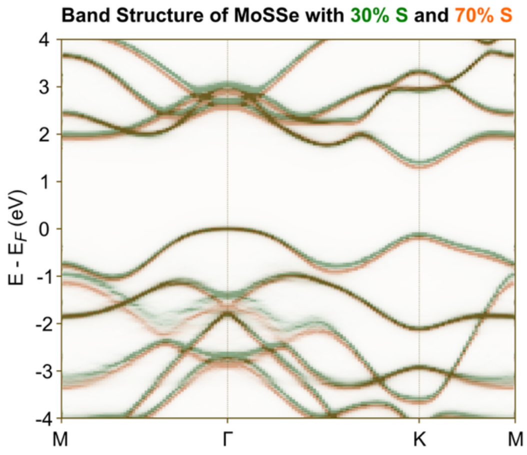

Figure S6 | DFT-Calculated Electronic Structure of Janus MoSSe with Varying Intrinsic Sulfur Dopant Concentrations.

Detailed DFT calculations comparing two models: a Janus MoSSe monolayer with 30% intrinsic substitutional sulfur (S) dopants on the top Se-layer (designated as 30% S) and one with 70% S. The plot overlays the calculated band structures for the 30% S (green) and 70% S (orange) models along the high-symmetry path (M-Γ-K-M), illustrating the impact of sulfur concentration on the electronic bands.

To ensure a fair comparison between the theoretically calculated DOS and our experimental STS spectra, we have applied a **distance-dependent correction** to the calculated DOS as shown in **Figure 4** of the main text. We reasoned that the calculated PDOS reflects the intrinsic properties of the material, whereas the STS measurement (d*I*/d*V*) captures the tunneling current originating from the out-of-plane orbitals of atoms at the surface. In our Janus MoSSe structure, the top-layer S and Se atoms are positioned approximately 1.7 Å closer to the STM tip than the atoms in the central Mo layer. Considering that the tunneling conductance (d*I*/d*V*) varies exponentially with the tip-sample distance[7-10] (proportional to exp(-2κz), where $\kappa = \sqrt{2m_{eff}\Phi}/\hbar$, where Φ is the work

function, and z is the tip-sample distance), the contribution from the more distant **Mo orbitals** will be significantly attenuated in the experimental measurement.

Based on our theoretical calculations, the work function is 5.48 eV for the 30% S sample and 5.21 eV for the 70% S sample, and the effective mass (m_{eff}) is 0.442 m_e, which are consistent with the previous literature[11-14]. We have used these parameters to apply a correction that appropriately scales the DOS contribution from the Mo orbitals. This correction allows the resulting DFT-calculated DOS to be more appropriately comparable with the experimentally acquired STS spectra.

References:

(1) Kresse, G.; Furthmüller, J. Efficient iterative schemes for *Ab initio* total-energy calculations. using a plane-wave basis set. *Physical Review B* **1996**, *54* (16), 11169–11186. DOI: 10.1103/physrevb.54.11169.

(2) Blöchl, P. E.; Först, C. J.; Schimpl, J. Projector augmented wave method:ab initio molecular. dynamics with full wave functions. *Bulletin of Materials Science* **2003**, *26* (1), 33–41. DOI: 10.1007/bf02712785.

(3) Perdew, J. P.; Burke, K.; Ernzerhof, M. Generalized Gradient Approximation Made Simple. *Physical Review Letters* **1996**, *77* (18), 3865–3868. DOI: 10.1103/physrevlett.77.3865.

(4) Peng, H.; Perdew, J. P. Rehabilitation of the Perdew-Burke-Ernzerhof generalized gradient. approximation for layered materials. *Physical Review B* **2017**, *95* (8). DOI: 10.1103/physrevb.95.081105.

(5) Wang, Z. 2H → 1T′ phase transformation in Janus monolayer MoSSe and MoSTe: an efficient. hole injection contact for 2H-MoS_2. *Journal of Materials Chemistry C* **2018**, *6* (47), 13000–13005. DOI: 10.1039/c8tc04951c.

(6) Cea, T.; Pantaleón, P. A.; Walet, N. R.; Guinea, F. Electrostatic interactions in twisted bilayer. graphene. *Nano Materials Science* **2022**, *4* (1), 27–35. DOI: 10.1016/j.nanoms.2021.10.001.

(7) Chen, C. J. Scanning tunneling microscopy: A chemical perspective. *Scanning microscopy.* **1993**, *7* (3), 4.

(8) Chen, C. J. Introduction to Scanning Tunneling Microscopy. **2007**. DOI: 10.1093/acprof:oso/9780199211500.001.0001.

(9) Chiu, M.-H.; Zhang, C.; Shiu, H.-W.; Chuu, C.-P.; Chen, C.-H.; Chang, C.-Y. S.; Chen, C.-H.; Chou, M.-Y.; Shih, C.-K.; Li, L.-J. Determination of band alignment in the single-layer MoS_2/WSe_2 heterojunction. *Nature Communications* **2015**, *6* (1), 7666. DOI: 10.1038/ncomms8666.

(10) Murray, C.; Jolie, W.; Fischer, J. A.; Hall, J.; Van Efferen, C.; Ehlen, N.; Grüneis, A.; Busse, C.; Michely, T. Comprehensive tunneling spectroscopy of quasifreestanding MoS_2 on graphene on Ir(111). *Physical Review B* **2019**, *99* (11). DOI: 10.1103/physrevb.99.115434.
(11) Chiu, Y.-P.; Huang, H.-W.; Wu, Y.-R. Utilizing the Janus MoSSe surface polarization in. designing complementary metal-oxide-semiconductor field-effect transistors. *Physical Review Applied* **2024**, *21* (4), 044046. DOI: 10.1103/physrevapplied.21.044046.
(12) Farkous, M.; El-Yadri, M.; Erguig, H.; Pérez, L. M.; Laroze, D.; Nguyen, C. V.; Binh, N. T. T.; Hieu, N. N.; Phuc, H. V.; Sadoqi, M.; et al. Anisotropy of effective masses induced by strain in Janus MoSSe and WSSe monolayers. *Physica E: Low-dimensional Systems and Nanostructures* **2021**, *134*, 114826. DOI: 10.1016/j.physe.2021.114826.
(13) Qin, X.; Wang, X.; Zhao, Y.; Ye, S.; Hilal, M.; Guo, J.; Zhang, W. InSb/Janus MoSSe van. der Waals heterostructure: First-principles calculation study of electronic structure and optical properties. *Solid State Communications* **2025**, *401*, 115921. DOI: 10.1016/j.ssc.2025.115921.
(14) Zhang, X.; Song, Y.; Zhang, F.; Fan, Q.; Jin, H.; Chen, S.; Jin, Y.; Gao, S.; Xiao, Y.; Mwankemwa, N.; et al. The Electronic Properties of Hydrogenated Janus MoSSe Monolayer: a First Principles Investigation. *Materials Research Express* **2019**, *6* (10), 105055. DOI: 10.1088/2053-1591/ab3ac8.

Supplementary Note 6

Defect types attribution

- **Type A defect**

As shown in our DFT simulations (**Figure S7**), selenium vacancies (V_{Se}) are significant structural defects that create undercoordinated Mo atoms. The dangling bonds from these atoms form new electronic states deep within the original bandgap, which is the dominant mechanism for the observed effective bandgap reduction.

These new defect states can then trap electrons, making the vacancy a localized charge center. However, the observed band edge movement (CBM down, VBM up) is not a simple electrostatic bending of the host material's bands. Instead, our STS measurement appears to be probing a spatial transition from the pristine MoSSe band structure to the smaller-gap electronic structure of the

defect itself and a more n-type characteristic resulting from local negative free charges induced by selenium vacancies.

In conclusion, we concur that the Type A defect's behavior is a collective effect: it is dominated by the formation of new states within bandgap from the V_{Se}, which then become charged and modulate the local electrostatics.

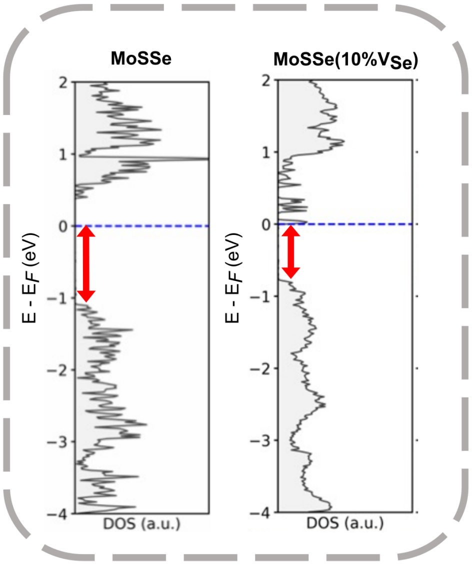

Figure S7 | DFT-Calculated Density of States Showing the Effect of Selenium Vacancies. Calculated density of states (DOS) for (**left**) pristine monolayer MoSSe and (**right**) MoSSe containing 10% selenium vacancies (V_{Se}). The introduction of V_{Se} results in the emergence of prominent in-gap states that effectively narrow the original bandgap. The red arrows highlight the effective bandgap in each case. This theoretical result provides strong support for identifying the experimentally observed Type A charge-trapping defect.

- **Type B defect**

Our hypothesis that Type B defects originate from the **plasma-induced structural disorder** is based on several converging lines of evidence. (1) First, as shown in a new high-resolution topography image (**Figure S8**), the area of the Type B defect exhibits a noticeably more disordered

surface morphology compared to the surrounding crystalline lattice. This STM morphological evidence provides a positively qualitative basis for our hypothesis. (2) Second, it is well-documented that plasma treatment can induce structural damage in 2D materials[1, 2], a point which is also supported by our own comparative Raman data (**Figure S4**), which suggests an increase in disorder after the conversion process. (3) Third, the electronic signature of the Type B defect—a significantly reduced local density of states consistent with an insulating scattering center—is analogous to the transition from conductive to insulating behavior observed in other material systems undergoing a crystalline-to-amorphous transformation[3].

Therefore, while we agree that alternative interpretations cannot be fully excluded without the complementary experiments discussed above, we believe "plasma-induced structural disorder" is the most plausible origin for the Type B defects.

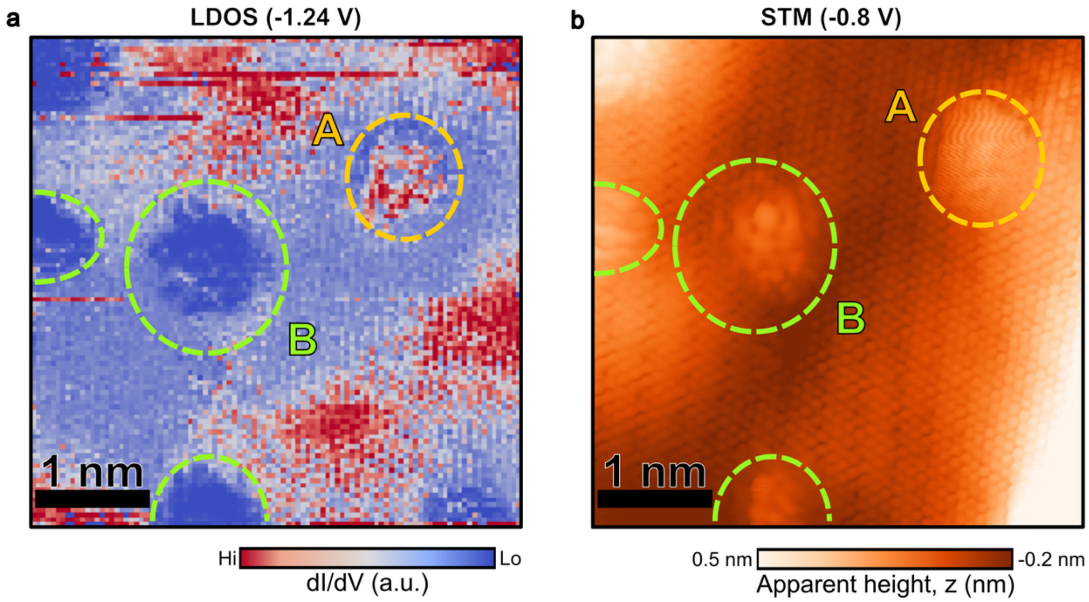

Figure S8 | Spatial Correlation between Defect Topography and Local Density of States.

Side-by-side comparison of an LDOS map and an STM topography image of the same region on the Janus MoSSe surface, demonstrating the direct real-space correspondence between a defect's physical appearance and its electronic signature. **(a)** Local density of states (LDOS) map acquired at a sample bias of -1.24 V, an energy corresponding to the in-gap states near the VBM. The color scale represents the d*I*/d*V* intensity, with red indicating high spectral weight (Hi) and blue indicating low spectral weight (Lo). **(b)** High-resolution STM topography of the same region acquired at -0.8 V. The direct comparison provides a clear visual correlation: the defect identified as Type A (yellow dashed circle) spatially corresponds to a region of high in-gap state density (bright/red feature in panel a). Conversely, the defect identified as Type B (green dashed circle) corresponds to a region of significantly suppressed in-gap state density (dark/blue feature in panel a). This provides strong, real-space evidence for the electronic classification of the two functionally distinct defect types.

References:

(1) Xiao, S.; Xiao, P.; Zhang, X.; Yan, D.; Gu, X.; Qin, F.; Ni, Z.; Han, Z. J.; Ostrikov, K. Atomic-layer soft plasma etching of MoS_2. *Scientific Reports* **2016**, *6* (1), 19945. DOI: 10.1038/srep19945.

(2) Kim, B. H.; Gu, H. H.; Yoon, Y. J. Atomic rearrangement of a sputtered MoS_2 film from. amorphous to a 2D layered structure by electron beam irradiation. *Scientific Reports* **2017**, *7* (1). DOI: 10.1038/s41598-017-04222-6.

(3) Shao, R.; Zheng, K.; Chen, Y.; Zhang, B.; Deng, Q.; Jiao, L.; Liao, Z.; Zhang, Z.; Zou, J.; Han, X. Direct observation of structural transitions in the phase change material $Ge_2Sb_2Te_5$. *Journal of Materials Chemistry C* **2016**, *4* (39), 9303–9309. DOI: 10.1039/c6tc01777k.

Supplementary Note 7

Statistical analysis of charge defect

We have performed a statistical analysis of the total charged defect density. These defects were identified based on their characteristic bias-dependent appearance in large-area STM topography images acquired at +1.5 V and -1.5 V. From analyzing multiple regions, each larger than 30 nm × 30 nm, we have determined the total density of these charged defects to be approximately $(5.05 \pm 2.36) \times 10^{11}$ cm^{-2}. The raw data used for this calculation, including the scanned area and the number of defects counted in each region, are summarized in **Table S2.**

Table S2 | Summary of data for statistical analysis of charged defect density.

**Region**	**Scan Area (nm × nm)**	**Number of defect**	**Defect density (cm^{-2})**
#1	65 × 65	14	3.31×10^{11}
#2	104 × 104	57	5.27×10^{11}
#3	65 × 65	37	8.74×10^{11}
#4	39 × 39	8	5.26×10^{11}
#5	104 × 104	29	2.68×10^{11}
